# Characterization of polymeric nanoparticles for treatment of partial injury to the central nervous system

**DOI:** 10.1016/j.dib.2016.02.019

**Published:** 2016-02-16

**Authors:** Ivan Lozić, Richard V. Hartz, Carole A. Bartlett, Jeremy A. Shaw, Michael Archer, Priya S.R. Naidu, Nicole M. Smith, Sarah A. Dunlop, K. Swaminathan Iyer, Matt R. Kilburn, Melinda Fitzgerald

**Affiliations:** a School of Chemistry and Biochemistry, The University of Western Australia, 35 Stirling Hwy, Crawley, WA 6009, Australia; b Experimental and Regenerative Neurosciences, School of Animal Biology, The University of Western Australia, 35 Stirling Hwy, Crawley, WA 6009, Australia; c Centre for Microscopy, Characterisation and Analysis, The University of Western Australia, 35 Stirling Hwy, Crawley, WA 6009, Australia

## Abstract

Before using nanoparticles for therapeutic applications, it is necessary to comprehensively investigate nanoparticle effects, both *in vitro* and *in vivo*. In the associated research article [1] we generate multimodal polymeric nanoparticles functionalized with an antibody, that are designed to deliver an anti-oxidant to astrocytes. Here we provide additional data demonstrating the effects of the nanoparticle preparations on an indicator of oxidative stress in an immortalized Müller cell line *in vitro*. We provide data demonstrating the use of nanoscale secondary ion mass spectroscopy (NanoSIMS) to identify specific ions in bulk dried NP. NanoSIMS is also used to visualize ^40^Ca microdomains in the *z* dimension of optic nerve that has been subjected to a partial optic nerve transection. The associated article [1] describes the use of NanoSIMS to quantify ^40^Ca microdomains in optic nerve from animals treated with various nanoparticle preparations and provides further interpretation and discussion of the findings.

## Specifications table

**Table:** 

Subject area	*Chemistry, Biology.*

More specific subject area	*Nanotechnology, Neuroscience.*
Type of data	*Graph, raw, images.*
How data was acquired	*Fluorescence microscopy (Nikon Eclipse Ti inverted microscope), nanoscale secondary ion mass spectroscopy (NanoSIMS-CAMECA NanoSIMS 50 ion microprobe at the University of Western Australia).*
Data format	*Data are both analyzed and raw.*
Experimental factors	*Cells were incubated with H*_*2*_*O*_*2*_*and various nanoparticle preparations prior to analysis of an oxidative stress indicator. Optic nerve tissue sections were prepared from cryopreserved optic nerve 24 h following partial optic nerve injury in adult PVG rat.*
Experimental features	*Immunoreactivity of carboxymethyl lysine in an immortalized Müller cell line was assessed in the presence of H*_*2*_*O*_*2*_*stress and the anti-oxidant resveratrol encapsulated within nanoparticles, relative to controls. NanoSIMS spectra of PGMA polymer nanoparticles containing magnetite were analysed to demonstrate spatial resolution. NanoSIMS analysis of cryopreserved injured optic nerve was used to show ^40^Ca microdomains in the z dimension.*
Data source location	*Perth, Australia.*
Data accessibility	*Data is with this article.*

## Value of the data

• The data provide measures of oxidative stress in stressed cells exposed to a free antioxidant, compared to the antioxidant encapsulated in nanoparticles (NP).
• A raw nanoscale secondary ion mass spectroscopy (NanoSIMS) spectra and spectral resolution data from dried NP containing magnetite are provided, showing how NanoSIMS can be used to detect NP.
• NanoSIMS data describing the distribution of Ca microdomains in the *z* projection in optic nerve following partial transection injury are also provided.
• NanoSIMS analyses of the distribution of Ca microdomains can be conducted following injury or in disease states, in order to detect changes that may be amenable to therapeutic intervention.

## 1 Data

The oxidative stress indicator carboxymethyl lysine was assessed in an astrocyte-like immortalized Müller cell line (rMC1 cells). Cells were stressed with H_2_O_2_ and exposed to free antioxidant and antioxidant encapsulated within NP. A sample raw NanoSIMS spectra of bulk dried NP and associated spatial resolution data demonstrate the ability to detect ^56^Fe within NP. NanoSIMS can be used to detect ^40^Ca microdomains in the *z* dimension of optic nerve following partial optic nerve transection.

## 2 Experimental design, materials and methods

**Fig. 1:**
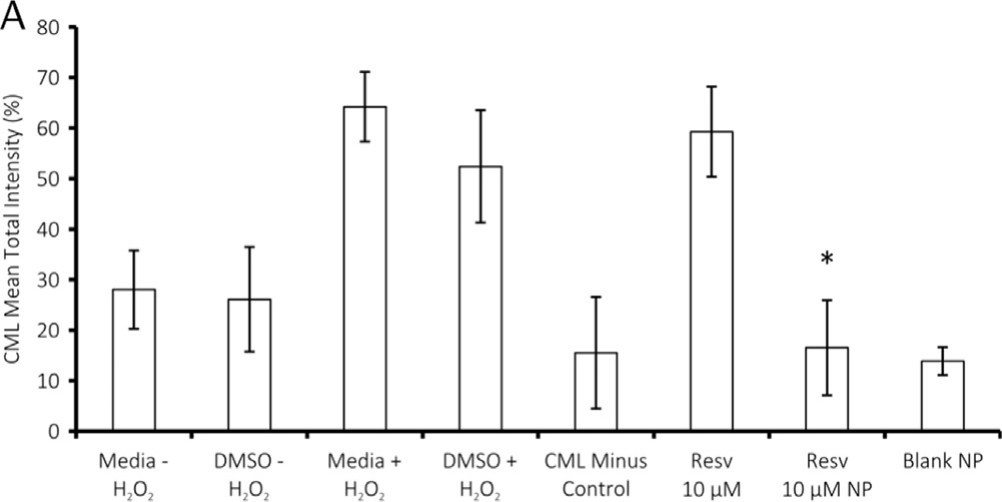
Cells were cultured for 48 h in Neurobasal media (with 10% foetal calf serum and 1% glutamax) in wells pre-coated sequentially with 100 μL poly-l-lysine (10 μg/mL) and laminin (100 μg/mL). Oxidative stress was induced in immortalized astrocyte rMC-1 cells through the addition of 5 mmol/L H_2_O_2_ in cell growth media. 10 μM Resveratrol or NP (200 μg/mL of NP; 10 μM of resveratrol) were added to cultures and incubated for a further 24 h. The concentration of nanoparticles was capped at a maximum of 200 µg/ml, above which they have been found to be toxic [2] and equivalent concentrations of resveratrol were delivered in free form or encapsulated in NP. Data are presented as CML mean total intensity ± S.E.M.; ∗=*p*<0.05 significantly different from DMSO vehicle with H_2_O_2_:DMSO+H_2_O_2_. 10 µM resveratrol within NP was more effective at reducing CML immunoreactivity than 10 µM free resveratrol. Note that empty NP (blank NP) were also effective at reducing CML immunoreactivity, as has been reported for other NP preparations [3]. The CML minus control describes immunoreactivity without the presence of the anti-CML antibody.

Comparison of the effects of free antioxidants and antioxidants within NP on mean total immunointensity of the oxidative stress indicator carboxymethyl lysine (CML) *in vitro*, assessed using ImageJ image analysis software (Fig. 1).

**Fig. 2:**
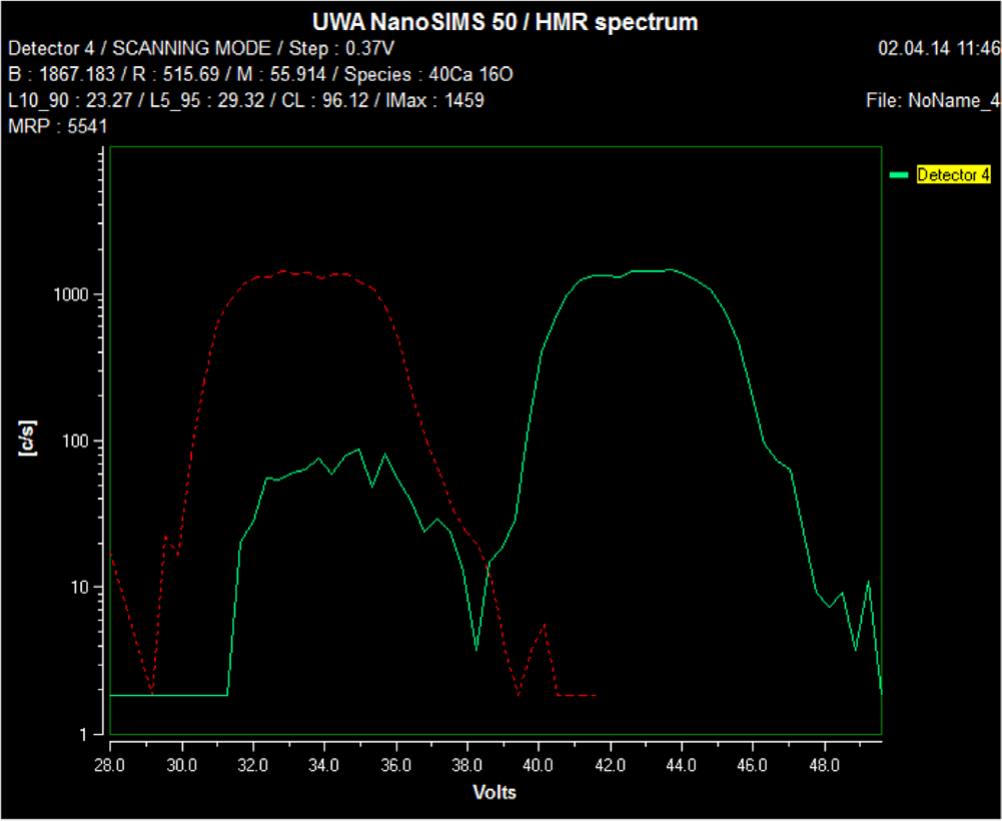
Sample spectrum produced during the tuning of NanoSIMS detector 4 at 56.00 u showing overlap of ^56^Fe and ^28^Si_2_ and ^40^Ca^16^O mass peaks when assessing bulk dried NP containing magnetite. NP were synthesized according to established procedures [1], [4]. The red peak is the Fe signal from metallic Fe used to calibrate peak position. The green peaks are acquired from the sample – the left-hand peak is Fe, while the right peak is a combination of the CaO and Si_2_ peaks. There is no significant overlap of the CaO/Si_2_ peak on the Fe peak.

Raw NanoSIMS spectra (Fig. 2) and spectral resolution data (Table 1) are shown to illustrate the nature of the data generated using the NanoSIMS and the ability to differentially detect specific ions.

**Table 1:** Spectral resolution at 56.00 u.

***Species symbol***	***Mass***	***Radius***
^56^Fe	*55.935*	*515.788*
^28^Si_2_	*55.954*	*515.876*
^40^Ca^16^O	*55.958*	*515.892*

Mass of secondary ion fragments at mass 56.00 u. Detector was successfully tuned to detect ^56^Fe and not ^28^Si_2_ and ^40^Ca^16^O.

**Fig. 3:**
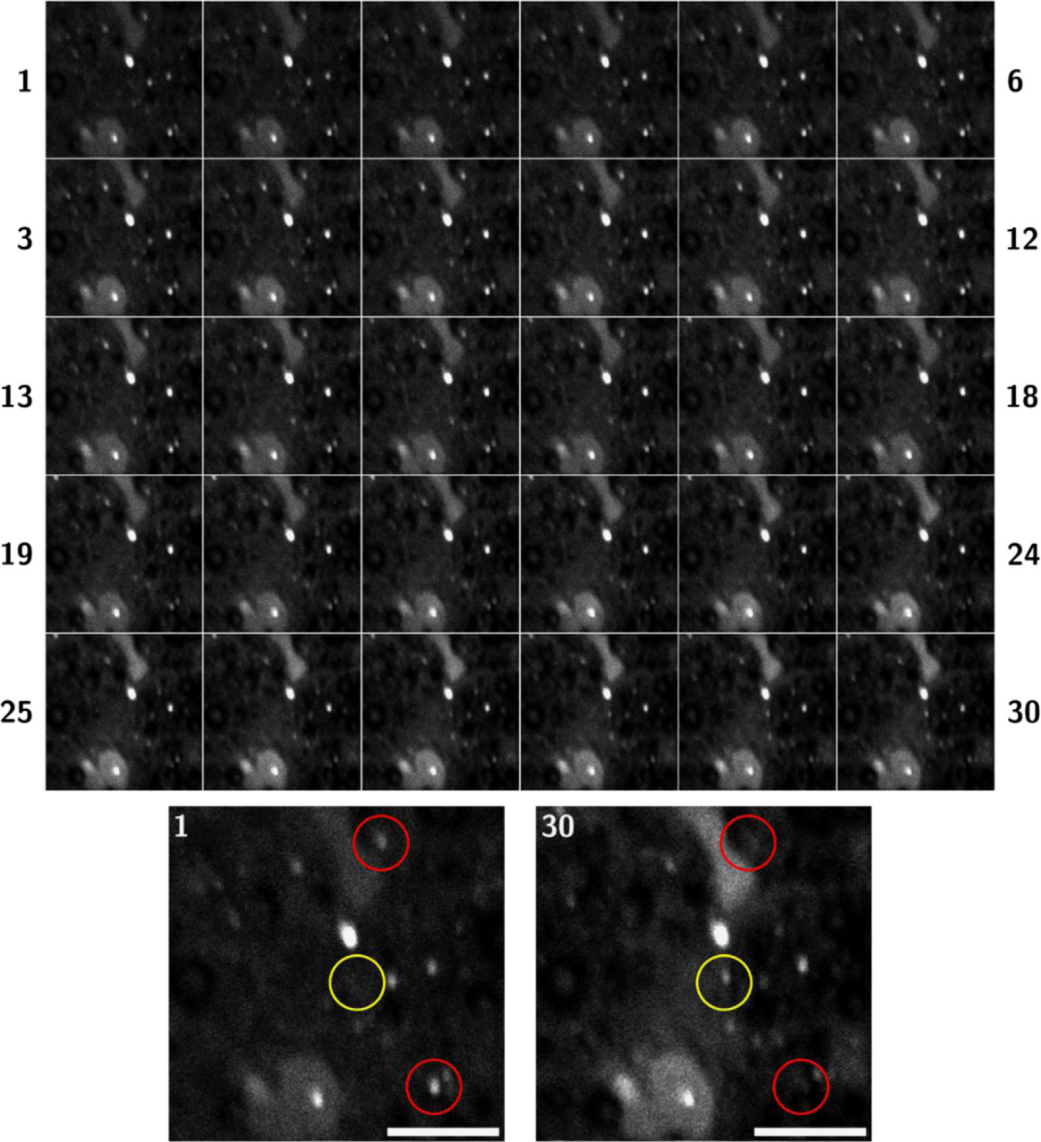
Partial optic nerve transection and cryopreservation of tissue was conducted as described, [5] tissue was collected 24 h following partial optic nerve injury. Images were collected with a resolution of 256×256 pixels, dwell time of 10 ms/px, 30 planes at 655.36 s/plane, for a total acquisition time of 16,695 s, all FOV 30×30 μm. Full details of image acquisition are provided in [1]. Top panel: a montage of each slice is presented in order from left to right. Bottom panel: occasional Ca microdomains were observed to disappear (red circles), and new Ca microdomains to appear (yellow circle) when scanning from slice 1–30; scale bar=10 μm.

Analysis of Ca distribution in the *z*-dimension of an optic nerve field of view (FOV) following partial optic nerve transection, collected using NanoSIMS. All procedures involving animals conformed to the National Health and Medical Research Council of Australia Guidelines on the Use of Animals in Research and were approved by the Animal Ethics Committee of The University of Western Australia (approval number RA3/100/1201) (Fig. 3).
